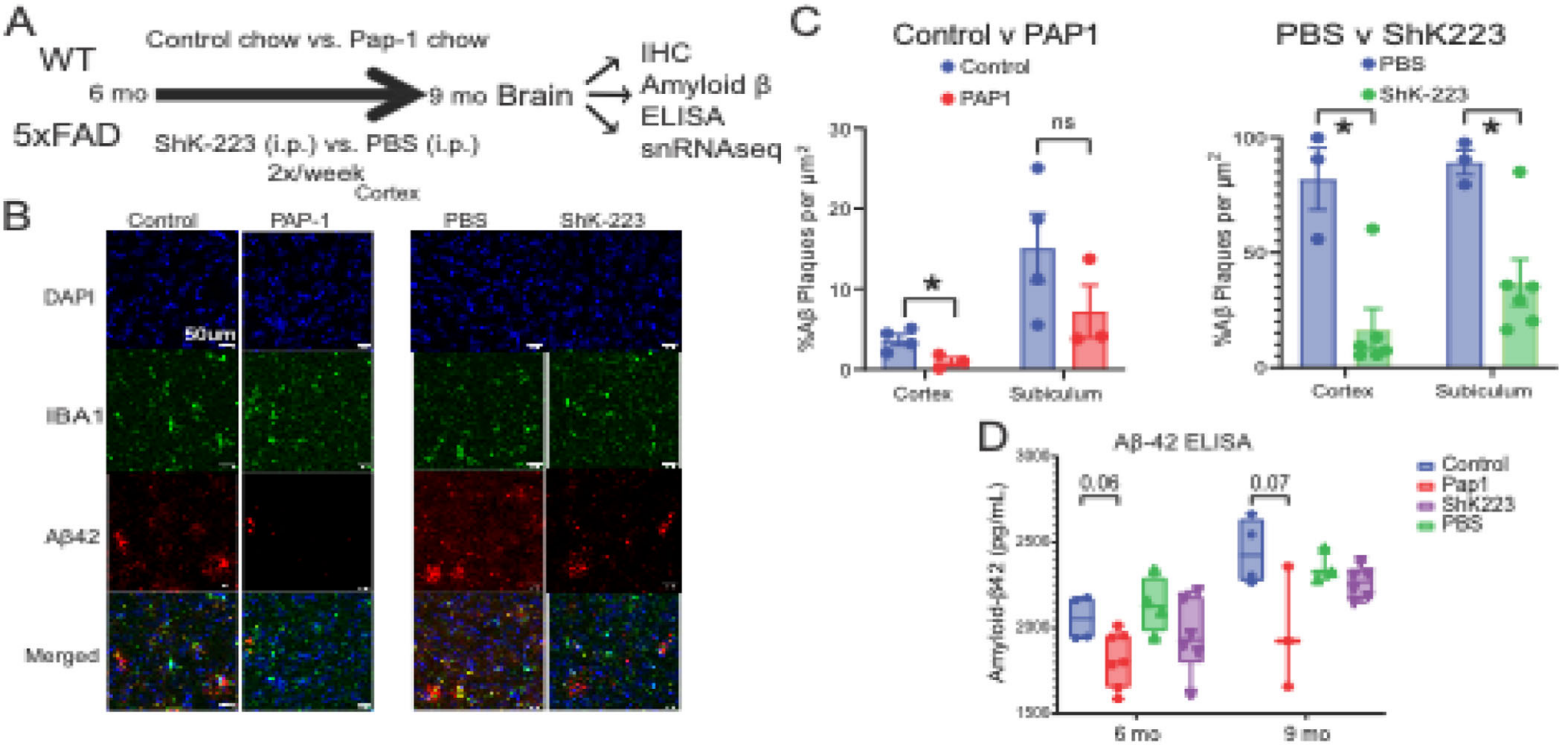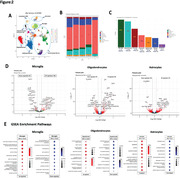# Neuropathological and cell type‐specific effects of Kv1.3 blockers in a pre‐clinical AD mouse model

**DOI:** 10.1002/alz.091970

**Published:** 2025-01-03

**Authors:** Upasna Srivastava, Christine A Bowen, Prateek Kumar, Maureen Sampson, Hailian Xiao, Hollis Zeng, Anson Sing, Caitlin Sojka, Tarun Bhatia, Heike Wulff, Steven A Sloan, Srikant Rangaraju, Department of Neurology

**Affiliations:** ^1^ Yale University School of Medicine, New Haven, CT USA; ^2^ Emory University, Atlanta, GA USA; ^3^ Emory University School of Medicine, Atlanta, GA USA; ^4^ University of California Davis, Davis, CA USA; ^5^ Center for Neurodegenerative Disease, Atlanta, GA USA

## Abstract

**Background:**

Kv1.3 channels are promising therapeutic targets to modulate neuroinflammatory responses in neurodegenerative disease including Alzheimer’s disease (AD). Although the ability of Kv1.3 blockers to reduce neuropathology in AD mouse models has been demonstrated, the cellular mechanisms remain unclear.

**Method:**

5xFAD mice aged 6 months were treated with PAP‐1 (a small molecule Kv1.3 blocker, chow), ShK‐223 (peptide blocker, intra‐peritoneal twice‐weekly), control chow, or vehicle injections until 9 months age. We assessed neurobehavior, Aβ burden neuropathology and single‐nuclei transcriptomics (10x Chromium 3’). For Sn‐RNA‐seq, 137,375 cortical nuclei from n = 4 mice/group passed QC after alignment, data integration (Seurat) and batch effects removal (Harmony), followed by cell‐type annotation using a reference and its prediction score, pseudo‐bulk analysis was performed using cell‐type subsets based on the contrast PAP‐1 versus Control. DEGs were identified using (glmGamPoi) based on log2FC > < 0.25 and adjP <0.05, and magnitude of drug effect was compared across cell types. Utilized GSEA with ClusterProfiler and enrichGO to investigate Kv1.3 blockade effects on diverse cell populations.

**Result:**

While both Kv1.3 blockers (PAP‐1 and ShK‐223) reduced Ab plaque burden in 5xFAD mice, lower Ab42 ELISA‐measured levels and improved fear conditioning behavior were only observed in the PAP‐1 group. snRNAseq revealed distinct cell type clusters, with consistent cell type proportions across all treatment groups. 5xFAD mice exhibited increased disease‐associated microglia compared to WT mice. In pseudo‐bulk analyses, PAP‐1 had the most significant impact on microglia compared to other cell types, evident from the proportion of DEGs in the sampled transcriptome. In microglia, PAP‐1 upregulated complement genes (C1qa, C1qb, C1qc), induced glial cell differentiation, transmembrane transport and voltage‐gated potassium channel. PAP‐1 treatment enhanced myelination (Plp1), oligodendrocyte differentiation and neuronal ensheathment in oligodendrocytes. These aligned with heightened activity of cognition and synapse‐related genes in astrocytes, glutamatergic and GABAergic neurons.

**Conclusion:**

Our studies re‐affirm beneficial neuropathological effects of pharmacological Kv1.3 blockade in Ab models, and additionally reveal differential effects and mechanisms of membrane permeable and brain penetrant versus non‐permeant Kv1.3 blockers. PAP‐1 prominently affects microglia and oligodendrocytes, suggesting they may be potential targets of Kv1.3 blockade. Additionally, evidence indicates pro‐myelination and synaptic‐protective effects of Kv1.3 blockade.